# Retrospective molecular investigation of Mayaro and Oropouche viruses at the human-animal interface in West-central Brazil, 2016–2018

**DOI:** 10.1371/journal.pone.0277612

**Published:** 2022-11-17

**Authors:** Helver Gonçalves Dias, Raquel Curtinhas de Lima, Luciana Santos Barbosa, Thiara Manuele Alves de Souza, Jessica Badolato-Correa, Laura Marina Siqueira Maia, Raquel da Silva Ferreira, Nilvanei Aparecido da Silva Neves, Michell Charlles de Souza Costa, Leticia Ramos Martins, Emerson Marques de Souza, Michellen dos Santos Carvalho, Alexandre de Araujo-Oliveira, William de Almeida Marques, Gilberto Sabino-Santos, Marcio Schafer Marques, Gabriel Carvalho de Macedo, Wesley Arruda Gimenes Nantes, Filipe Martins Santos, Claudia Coutinho Netto, Thais Oliveira Morgado, Mateus de Assis Bianchini, Sandra Helena Ramiro Correa, Júlia Ramos de Almeida, Larissa Pratta Campos, Isabelle Marino de Souza, Wanessa Teixeira Gomes Barreto, Grasiela Porfírio, Jeronimo Augusto Fonseca Alencar, Heitor Miraglia Herrera, Renata Dezengrini Shlessarenko, Rivaldo Venancio da Cunha, Elzinandes Leal de Azeredo, Stephanie J. Salyer, Nicholas Komar, Alex Pauvolid-Corrêa, Flávia Barreto dos Santos

**Affiliations:** 1 Laboratório de Imunologia Viral, Instituto Oswaldo Cruz, Fundação Oswaldo Cruz (Fiocruz), Rio de Janeiro, Brazil; 2 Laboratório de Genética, Instituto de Puericultura e Pediatria Martagão Gesteira (IPPMG), Universidade Federal do Rio de Janeiro (UFRJ), Rio de Janeiro, Brazil; 3 Laboratório de Virologia, Faculdade de Medicina, Universidade Federal de Mato Grosso (UFMT), Cuiabá, Brazil; 4 Laboratório de Diptera, Instituto Oswaldo Cruz, Fiocruz, Rio de Janeiro, Brazil; 5 Center for Virology Research, Ribeirão Preto Medical School University of São Paulo, Ribeirão Preto-SP, Brazil; 6 Department of Tropical Medicine, Tulane University School of Public Health and Tropical Medicine, New Orleans-LA, United States of America; 7 Laboratório de Biologia Parasitária, Programa de Pós-Graduação em Ciências Ambientais e Sustentabilidade Agropecuária, Universidade Católica Dom Bosco, Campo Grande, Brazil; 8 Centro de Reabilitação de Animais Silvestres (CRAS), Campo Grande, Brazil; 9 Hospital Veterinário, Universidade Federal de Mato Grosso (UFMT), Cuiabá, Brazil; 10 Faculdade de Medicina Veterinária, Universidade Federal de Mato Grosso (UFMT), Cuiabá, Brazil; 11 Laboratório de Ecologia de Populações e do Movimento, Programa de Ecologia e Conservação, Universidade Federal de Mato Grosso do Sul (UFMS), Campo Grande, Brazil; 12 Fiocruz, Campo Grande, Mato Grosso do Sul, Brazil; 13 Departamento de Clínica Médica, Universidade Federal do Mato Grosso do Sul, Campo Grande (UFMS), Campo Grande, Brazil; 14 Division of Global Health Protection, Center for Global Health, U.S. Centers for Disease Control and Prevention (CDC), Atlanta, Georgia, United States of America; 15 Arboviral Diseases Branch, Division of Vector-borne Diseases, U.S. Centers for Disease Control and Prevention (CDC), Fort Collins, Colorado, United States of America; 16 Departamento de Veterinária, Universidade Federal de Viçosa, Viçosa, MG, Brazil; University of Texas Medical Branch at Galveston, UNITED STATES

## Abstract

Mayaro virus (MAYV, Togaviridae) and Oropouche orthobunyavirus (OROV, Peribunyaviridae) are emerging enzootic arboviruses in Latin America. Outbreaks of febrile illness associated with MAYV and OROV have been reported among humans mainly in the northern region of Brazil since the 1980s, and recent data suggest these viruses have circulated also in more populated areas of western Brazil. MAYV shares mosquito vectors with yellow fever virus and it has been historically detected during yellow fever epidemics. Aiming to investigate the transmission of OROV and MAYV at the human-animal interface during a yellow fever, chikungunya and Zika outbreaks in Brazil, we conducted a retrospective molecular investigation in 810 wild and domestic animals, 106 febrile patients, and 22.931 vectors collected from 2016 to 2018 in Cuiaba and Campo Grande metropolitan regions, western Brazil. All samples tested negative for OROV and MAYV RNA by RT-qPCR. Findings presented here suggest no active circulation of MAYV and OROV in the sampled hosts. Active surveillance and retrospective investigations are instrumental approaches for the detection of cryptic and subclinical activity of enzootic arboviruses and together serve as a warning system to implement appropriate actions to prevent outbreaks.

## 1. Introduction

Epidemic arthropod-borne viruses (arboviruses) such as dengue (DENV), Zika (ZIKV), and chikungunya (CHIKV) cause a significant public health burden throughout the globe. Arboviruses are spread across different regions of the world and have a major health impact on populated areas of Latin America [1, 2]. Brazil is one of the world’s most affected countries, and the West-Central region of the country has been severely hit by arbovirus epidemics in the last decade [3–5]. Enzootic arboviruses, such as yellow fever virus (YFV), have great potential for emergence and urbanization, requiring special attention from one-health surveillance systems. The neglected Mayaro virus (MAYV, *Togaviridae*) and Oropouche orthobunyavirus (OROV, *Peribunyaviridae*) are a great concern, as they have been historically involved in outbreaks in rural communities of the Amazon region, and more recently been suggested to be involved in sporadic febrile cases in the West-central and northeast regions of Brazil [6–12].

MAYV, one of the nine enzootic alphaviruses reported in Brazil, can cause acute febrile illness with headache and marked arthralgia [13]. After CHIKV, which is involved in urban epidemics, MAYV is the most important cause of human illness due to alphaviruses in the country [7, 14, 15]. Maculopapular rash is seen in a great proportion of patients with mayaro fever, and arthralgia can become chronic and persist for about 2–3 months [14, 15]. MAYV is believed to be primarily maintained in enzootic cycles of transmission involving mainly non-human primates such as the Silvery (*Mico argentatus*), Santarem (*Mico humeralifer*), and White (*Mico leucippe*) marmosets, and acrodendrophilic mosquito species as *Haemagogus janthinomys*, which is also the main vector of YFV in Brazil [7]. Several other studies have demonstrated that not only other species of vertebrates, but also vectors have been found infected and can potentially participate in MAYV cycles of transmission [16–19]. Because MAYV and YFV share the same main vector and have a similar enzootic cycle of transmission, MAYV has been historically detected during yellow fever outbreaks reported in the north region of the country [7].

Clinical infection caused by OROV is characterized by an abrupt onset and fever, headache, myalgia, arthralgia, dizziness, chills and photophobia [14]. Oropouche fever has a high attack rate and large outbreaks have been reported in Brazil [20]. A portion of patients present clinical recurrence of symptoms after an initial period of improvement [9, 14, 15, 21]. OROV-associated neurological disease, especially aseptic meningitis, has been reported in immunocompromised individuals, and more recently in healthy individuals [14, 22–25]. OROV has unclear and apparently complex cycles of transmission involving different ecological niches with different species of vertebrates and hematophagous arthropods acting as amplifying hosts and vectors, respectively. It is suggested that OROV is maintained in sylvatic cycles involving sloths, such as *Bradypus tridactylus* and non-human primates, including *Callithrix* spp. as amplifying hosts, and mosquito species *Aedes serratus* and *Coquillettidia venezuelensis* as vectors [26, 27]. Additionally, outbreaks of oropouche fever in rural villages have been attributed to epidemic transmission between humans and *Culicoides paraensis* midges and *Culex quinquefasciatus* mosquitoes, which is corroborated by the large number of virus isolations and molecular detection from blood samples of affected patients [9, 15, 28].

For both MAYV and OROV, birds are believed to eventually act as secondary amplifying hosts [17, 29–31]. Several studies have demonstrated, by serological methods, that other groups of vertebrates, including wild and domestic species are eventually exposed to both arboviruses [16, 32–34]. Studies focused on the potential capacity of these vertebrates to present viremia and ultimately potential role in enzootic cycles of transmission of MAYV and OROV have been less reported. The west-central Brazil, especially the metropolitan regions of Cuiabá and Campo Grande, have socio-economic, environmental and climatic characteristics favorable to the proliferation of arboviruses. This region has been historically affected by arbovirus epidemics and in recent years silent circulation of MAYV and OROV has been reported. The main objective of the present study is to investigate OROV and MAYV in samples from humans, wild and domestic vertebrates, and mosquitoes collected during epidemics of ZIKV, CHIKV and YFV between 2016 and 2018 at the human-animal interface in West-Central Brazil.

## 2. Materials and methods

### 2.1 Ethics statement

This study was approved by the research ethics committee, and the Ethics Committee of Plataforma Brasil, FIOCRUZ (CAAE 57221416.0.1001.5248). All subjects were informed about the research and signed a written consent document. Biological samples were also reported to the National System for Access to Genetic Heritage and Associated Traditional Knowledge (SISGEN) according to the Law 13.123/2015 and Decree 8772/2016. Animal sampling was approved by the U.S. Centers for Disease Control and Prevention Institutional Animal Care and Use Committee (Protocol number 2808SALMULX-A2).

### 2.2 Sampling sites

Sampling was performed between 2016 and 2018 in different sites in urban and peri-urban areas of two cities from two different states of Brazil: Cuiabá, state of Mato Grosso (MT) and Campo Grande, state of Mato Grosso do Sul (MS). Geographical coordinates of all samples, including animals, mosquitoes, and humans, are available as supplementary information, as previously described [35, 36]. For the map construction, collection subsites were geo-referenced and the shape files were extracted from the open access (public domain) cartographic base of Brazilian Institute of Geography and Statistics (IBGE) accessed at https://www.ibge.gov.br/geociencias/downloads-geociencias.html.

### 2.3 Mosquito sampling

To collect different species of mosquitoes, we used multiple traps types, including CDC light traps, Insectazooka or Prokopak aspirators, and BG-sentinel traps in several types of microhabitats in each study site. Urban and peri-urban species, including *Ae*. *aegypti*, *Cx quinquefasciatus* and sylvatic species such as *Sabethes* spp. and *Haemagogus* spp. were sampled (Table 1). After collection, the mosquitoes were kept frozen (-70°C) and sent to the laboratory, where they were later identified, keeping the cooling to preserve the viral RNA and the integrity of the material. Mosquitoes were organized into pools of up to 25 individuals, according to species, sex, place and date of collection and type of trap.The mosquito sampling procedures, as well as the previous molecular testing conducted with all these samples showing that none of them was positive for ZIKV RNA were previously described [35].

**Table 1:** Non-human vertebrates and mosquitoes sampled in central-western Brazil between 2017 and 2018 and tested for OROV and MAYV, by state. MT, state of Mato Grosso; MS, state of Mato Grosso do Sul.

	MT	MS		MT	MS
Vertebrate Species	N	N	Mosquito Species	N	N
**Domestic**			*Culex* spp.	19,512	1,656
*Bos indicus/taurus*	64	112	*Wyeomyia* sp.	307	6
*Canis lupus familiaris*	63	111	*Aedes aegypti*	232	48
*Equus ferus caballus*	61	99	*Psorophora dimidiata*	80	111
*Felis silvestris catus*	36	49	*Culex nigripalpus*	175	0
**Wild**			*Psorophora albigenu*	121	13
*Nasua nasua*	4	79	*Aedes scapularis*	14	87
*Didelphis albiventris*	57	16	*Aedes albopictus*	63	31
*Mico melanurus*	29	0	*Psorophora* spp.	60	34
*Sapajus cay*	0	11	*Culex quinquefasciatus*	75	0
*Callithrix jacchus*	0	4	*Aedes* spp.	21	41
*Sapajus apella*	5	0	*Haemagogus janthinomys*	6	46
*Didelphis aurita*	3	0	*Psorophora cingulata*	5	34
*Alouatta caraya*	0	3	*Psorophora cilipes*	1	37
*Aotus lemurinus*	2	0	*Mansonia* sp.	32	2
*Ateles marginatus*	2	0	*Psorophora lanei*	10	22
			*Haemagogus leucocelaenus*	20	2
			*Sabethes* spp.	1	15
			*Haemagogus* sp.	11	0

### 2.4 Wild and domestic animals sampling

Animal sampling targeted abundant domestic species including horses, cattle, dogs and cats, as well as free-ranging and captive peri-urban synanthropic wildlife species including opossums, coatis and non-human primates (Table 1). Domestic animal sampling was conducted primarily in residential neighborhoods, shelters, ranches, state police equine facilities and equestrian societies. For the sampling of wildlife, campuses of local universities, veterinary hospitals, residential neighborhoods and nature reserves were used as sampling locations. A whole blood sample was collected from each animal by venipuncture and placed in a tube with anticoagulant (sodium citrate). When enough volume was available, plasma was separated from blood samples by centrifugation. Although whole blood and plasma samples were collected from all animals and kept frozen (-70°C) until viral RNA extraction, only whole blood was used in the present study. The vertebrate sampling procedures, as well as the previous molecular testing conducted with all these samples, showing that none of them was positive for ZIKV RNA, were previously described [35]. RNA samples were thawed only once for initial testing for ZIKV and here we use the same samples for the investigation of OROV and MAYV.

### 2.5 Human clinical sampling

We selected 106 clinical samples collected from February to March 2016 from acute febrile patients (onset of disease ≤7 days) attended at the Walfrido Arruda Emergency Care Unit, Campo Grande, MS, Brazil. No human samples were collected in MT. Blood samples were collected, allowed to coagulate at ambient temperature, and centrifuged for separation of serum, which was stored at −70°C. In addition to the investigation carried out here, these serum samples were previously tested for ZIKV, CHIKV and DENV, and results reported elsewhere [36].

### 2.6 Real-Time Reverse Transcriptase Polymerase Chain Reaction (RT-qPCR) for Mayaro and Oropouche viruses

Viral nucleic acid was extracted from whole blood samples of animals, triturated mosquito pools and serum of human samples using the ZR-Viral RNA or DNA/RNA kits (Zymo Research, Irvine, CA, USA) according to the manufacturer’s instructions. Extracted RNA/DNA was subjected to a specific TaqMan duplex RT-qPCR method for the detection of a region of non-structural protein 1 (NSP1) gene of MAYV and of segment S of OROV, as previously described [37]. The RT-qPCR used is also capable of detecting Oropouche-like viruses that carry the OROV S segment. We used the GoTaq® Probe 1-Step RT-qPCR (Promega) for amplification with the recommended cycling parameters. RNA (5 μL) sample was used as a template in a reaction final volume of 25 μL, and all assays were performed using the *7500* Real-Time PCR Systems (Applied Biosystems). RT-qPCR was performed using positive (MAYV ATCC VR 66, strain TR 4675, GenBank #MK070492 and OROV strain BeAn 19991, GenBank accession #KP052852.1, #KP052851.1, #KP052850.1) and negative viral controls for both viruses and we considered Ct value lower than 38 as positive with a limit of detection between two and 20 copies per reaction (mean Ct values, 28.9 and 31.2 for MAYV and OROV, respectively).

## 3. Results

A total of 595 domestic animals from four species, 215 wild mammals from 12 species, 106 human serum samples of acute febrile cases, and 22,931 specimens of mosquitoes of 37 species were tested for OROV and MAYV by RT-qPCR.

**Fig 1:**
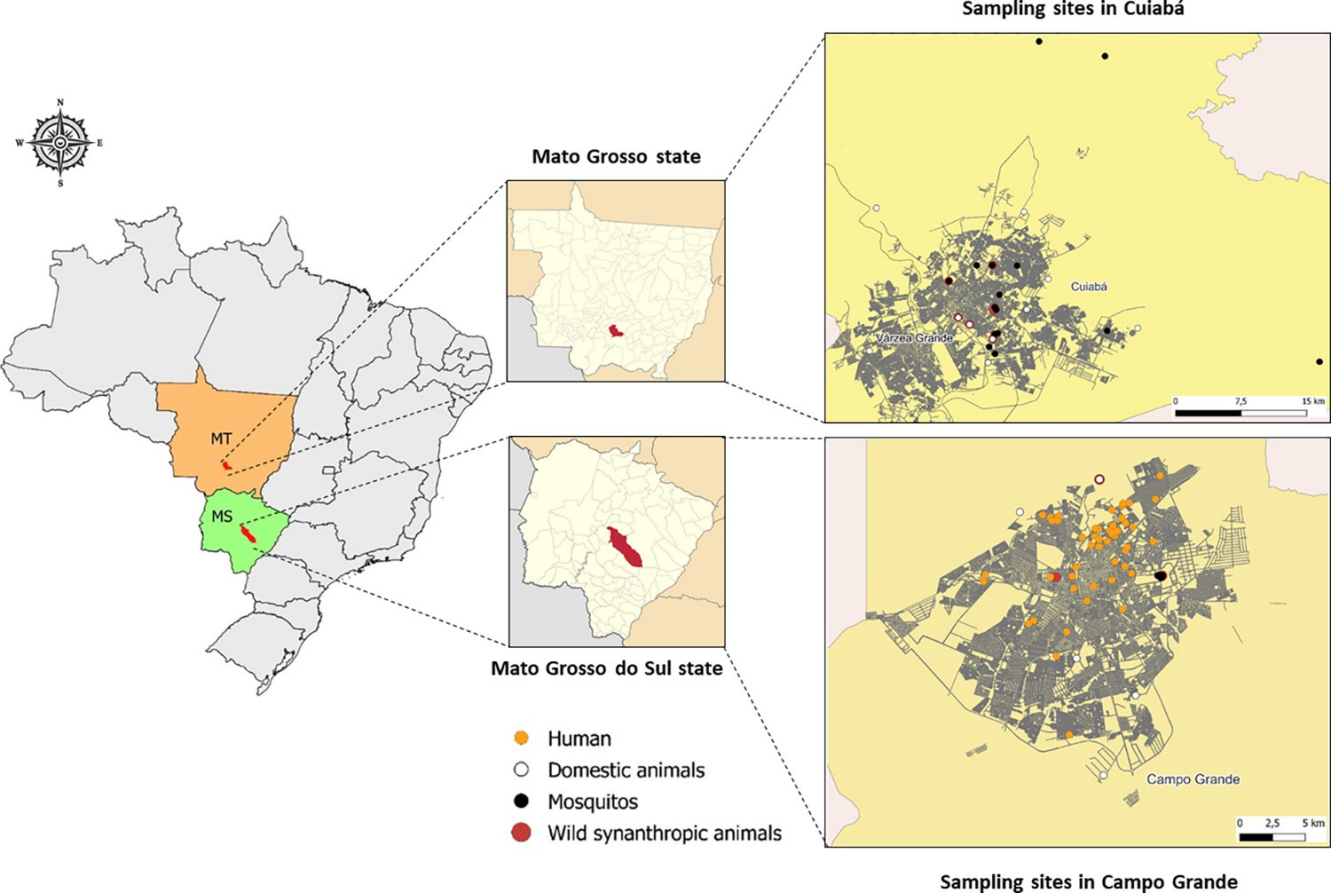


The sampling strategy for vertebrates and mosquitoes was previously described, and non-human vertebrates and mosquitoes were collected in 30 sub-sites of the state of Mato Grosso (MT) and 14 sub-sites of the state of Mato Grosso do Sul (MS) (Fig 1).

Domestic species tested in both states were: *Bos indicus/taurus* (*n* = 176), *Canis lupus familiaris* (*n* = 174), *Equus ferus caballus* (*n* = 160) and *Felis silvestris catus* (*n* = 85). Regarding wild animals, the three species with the highest number of individuals were: *Nasua nasua* (*n* = 83), *Didelphis albiventris* (*n* = 73) and *Mico melanurus* (*n* = 29) (Table 1).

A total of 22,931 adult mosquitoes from MT and MS, belonging to 37 species and distributed in 951 pools were tested for OROV and MAYV. A total of 6,603 specimens were non-engorged females and 16,328 were males. *Culex* spp. (*n* = 21.418; 93,4%) was the most sampled genera, followed by *Aedes* spp. (*n* = 537; 2,3%), *Psorophora* spp. (*n* = 528; 2,3%), *Wyeomyia* spp. (*n* = 313; 1,3%), *Haemagogus* spp. (*n* = 85; 0,4%), *Mansonia* spp. (n = 34; 0,15%), and *Sabethes* spp. (*n* = 16; 0,07%).

Human samples of cases of acute febrile illness were all from the city of Campo Grande, MS. A total of 106 samples were tested, being 69 (63,9%) female and 38 (36,1%) male, both sex with a medium age of 34 years. All vertebrate and mosquito samples included in this study tested negative for MAYV and OROV.

## 4. Discussion

The RNA used in the present study was extracted and used primarily for the detection of ZIKV in mosquitoes and whole blood of animals, as previously mentioned. Aware that improper and excessive handling of samples after collection can have an effect on RNA degradation, we found opportune the fact that the samples were thawed only once (then kept frozen at -70°C) and tested them for MAYV and OROV with all the necessary precautions to avoid degradation.

The negative RT-qPCR results presented here suggest no viremia caused by MAYV or OROV during the sampling period in the domestic and wild species of vertebrates from Cuiaba and Campo Grande metropolitan areas. Even using a very sensitive RT-qPCR multiplex protocol, which is capable of detecting a low number of RNA copies, the samples were negative.

All sera from humans with acute febrile illness were also negative for OROV and MAYV RNA. Of the 106 human samples tested, 42 were positive by RT-qPCR for DENV and/or ZIKV, as previously described [36], suggesting that febrile patients from Campo Grande were exposed to the most common epidemic arboviruses as DENV and ZIKV other than MAYV and OROV.

The results described here do not align with previous studies in humans temporally and/or geographicaly related [38–41], but previous studies have reported evidence of MAYV infection in humans from other areas of MT and MS [16, 34, 42, 43]. Additional molecular and serological evidences of MAYV have also been reported in the neighboring state of Goiás (GO), corroborating the circulation of MAYV in central-western Brazil [44–46]. These evidences highlight the need for differential diagnosis for MAYV and OROV in acute human febrile cases suspected of DENV, ZIKV or CHIKV infections in central-western Brazil. Notably, a growing number of studies have reported the detection of MAYV during outbreaks of epidemic arboviruses as DENV in Brazil and elsewhere [47–49].

One of the limitations of the present study is the non use of serological methods to investigate MAYV and OROV circulation in humans, wildlife and domestic animals. Antibody detection is instrumental for investigating infections for which the pathogen may be detected ephemerally by molecular methods while the resulting antibodies may be detected for the remaining life of the host. In a previous investigation of ZIKV conducted with the same non- human vertebrates and vectors, all samples tested negative for ZIKV RNA, but some domestic animals presented specific neutralizing antibodies for ZIKV [35]. Antibodies for MAYV and OROV have been found in patients and domestic animals from other areas of the same region, and also in other regions of the country [16, 32, 34, 44, 50, 51].

Regarding wildlife, evidence of MAYV and OROV circulation in non-human primates has been reported in MS, and from GO where an *Alouatta caraya* individual presented antibodies for OROV [27, 52–54]. In a study carried out with primates in French Guiana, 66% of the Colombian red howler monkey (*Alouatta seniculus*) and 18% of the Golden-headed tamarin (*Saguinus midas*) had antibodies for MAYV [17, 18].

In the present study, we investigated by RT-qPCR the circulation of MAYV and OROV in 56 non-human primates, including *Mico* and *Callithrix* marmosets and howler monkey, which are potential amplifying hosts. Of these, 29 were free-ranging Black-tailed marmoset (*Mico melanurus*), which was the most common non-human primate observed in the public parks sampled in the metropolitan area of Cuiaba, MT. The exposure of this species to arboviruses in the region is unknown [55, 56].

Regarding Campo Grande, MS, common marmoset (*Callithrix jacchus*), Hooded cappuchin (*Sapajus cay*) and black howler monkey (*Alouatta caraya*) were the most common non-human primates sampled and most of them were captive. Despite potential candidates for OROV and MAYV amplification, all individuals were negative by RT-qPCR which may be explained by either the absence of active infection in sampled animals or by the short viremia making unreliable a proper evaluation in the absence of complementary serological investigation [35, 54, 57]. The limited number of individuals tested from each species may also have influenced the results. The prevalence of exposure of non-human vertebrates to OROV and MAYV in the region is unknown and low prevalences may require greater number of individuals tested for detection.

Regarding vectors, the mosquito species that would be potentially involved in the transmission of MAYV and OROV at the human-animal interface in western Brazil remain unknown. *Haemagogus* spp., which are the main vector of MAYV, are diurnal acrodendrophilic mosquitoes and are more active in forested areas [7, 58]. Transmission in more urbanized areas could be hypothetically related to either sylvatic species in the genera *Haemagogus* and *Sabethes* reaching human settlements or by vectors that are fairly or very adapted to more urbanized environments, such as *Ae albopictus*, *Ae*. *aegypti* and *Cx*. *quinquefasciatus*.

In the present study, 85 individuals of *Haemagogus* were captured by different methods, including aspiration in tree canopies [35]. Of these, most were *Haemagogous janthinomys*, which is the most important vector for MAYV and all of them tested negative for both viruses. Also negative were 21,000 *Culex* mosquitoes, which included 75 *Cx*. *quinquefasciatus*, which is considered a secondary urban vector for OROV [38, 59, 60]. Evidence of arbovirus circulation in the absence of epidemics and epizootics frequently relies on the detection or isolation of arboviruses from vectors and sporadic cases [22, 39, 40, 43]. In the present study, all mosquito samples tested negative by molecular methods for OROV and MAYV.

These findings suggest absence of active circulation of both enzootic viruses in the mosquito population assessed in both cities. Despite some reports of OROV and MAYV in urban vector species in Brazil [38, 40, 46], the transmission and maintenance of these arboviruses by these anthropophilic species remains unclear in the country. Some vector competence studies have already shown that *Ae*. *albopictus* and *Cx*. *quinquefasciatus* are not efficient vectors for OROV under laboratory conditions [59–61]. Similar experimental studies using different populations of *Ae*. *Aegypti* and *Cx*. *quinquefasciatus* with MAYV have demonstrated that transmission by these species of vectors requires a high viremia, a feature not usually observed in human MAYV infections. These data suggest the vector competence of these urban vectors for MAYV transmission is limited [62–64].

The active surveillance reported here suggests low or absent circulation of MAYV and OROV in vertebrate and mosquito samples collected in MT and MS between 2016 and 2018. Active surveillance and retrospective investigations are instrumental approaches for the detection of enzootic arboviruses and together serve as a warning system to implement appropriate actions to prevent outbreaks.

## 5. Conclusions

Despite the absence of molecular evidence of MAYV and OROV in hundreds of vertebrate and vector samples reported here, we stress the importance of active surveillance at the human-animal interface in order to detect cryptic activity of neglected and emerging arboviruses in Brazil, as a warning system to implement appropriate actions to reduce outbreaks.

## Supporting information

S1 Table Sampling sites information. Additional information and geographic coordinates of sampling sites.
(XLSX)
